# Strategic outsourcing's role in driving economic value by examining mediating role of organizational capabilities and sustainable innovation

**DOI:** 10.3389/fpsyg.2022.933507

**Published:** 2022-07-28

**Authors:** Lei Shi

**Affiliations:** ^1^School of Marxism, Shanghai University of Finance and Economics, Shanghai, China; ^2^School of Economics and Management, Anhui Polytechnic University, Wuhu, China

**Keywords:** human resource outsourcing, organizational sustainability, economic sustainability, sustainable innovation, organizational capabilities

## Abstract

The study's overarching purpose was to investigate the impact of strategic HR outsourcing on organizational sustainability. This study also attempted to evaluate the function of organizational capabilities and HR proficiency as a mediator in the relationship between strategic HR outsourcing and organizational sustainability. Data was collected from 400 HR professionals in China using a questionnaire technique. The Smart-PLS software and a structural equation modeling (SEM) technique were used to evaluate the data. Organizational sustainability was found to be insignificantly related to strategic HR outsourcing. Furthermore, organizational capabilities and HR proficiency were discovered to mediate the association between strategic HR outsourcing and organizational sustainability. By analyzing the impact of strategic HR outsourcing on organizational sustainability, this paper provided an important theoretical contribution. In terms of practical applications, this article would surely help HR professionals to maintain and develop policies to encourage employees to be engaged and perform well. Furthermore, this study might be effective in resolving difficulties linked to organizational sustainability. The small sample size of this study, which included solely HR professionals working in Chinese firms, was one of its limitations. In addition, future studies can incorporate other constructs to acquire a deeper knowledge of the factors that influence employee health.

## Introduction

In the contemporary setting, businesses must contend with the characteristics of a corporate environment. The corporate environment of enterprises needs fast technological development, a brief life cycle, and global outreach. Organizations are now concentrating on integrating sustainability into corporate decision-making processes along with typical business concerns (Calabrese et al., 2019). The organizations can address environmental, societal, and socioeconomic issues by integrating sustainability with other work activities (Calabrese et al., 2019). Technology-driven organizations must be more creative and innovative to obtain a competitive advantage. To optimize the returns on investments, organizations are reinventing the methods of providing services and products globally. These organizations are also working on innovations to speed up the processes (Isah et al., 2017).

This shift is observed due to the apparent scarcity of resources and ever-increasing competition in the market. The demand of enterprises in less developed nations is to assess underlying overheads. This demand is based on the goal of implementing such operational models which are less expensive and more sustainable. This idea arose in response to currency rate instability, rampant inflation, and inconsistent policy decisions (Isah et al., 2017). Organizations are seeking continual improvement, re-engineered business operations, and coordinated supply chains to achieve this in the current globalization period (Isah et al., 2017). Organizations are constantly obligated to focus on improving their performance and achieve a competitive edge in any way possible. This is happening due to ever-increasing competition among the organizations of the same cadre. The business environment in China is also becoming hostile amidst a slew of laws and governmental interference (Marquis et al., 2015).

To achieve this aim, several business organizations have sought ways to increase performance outside of their companies' usual bounds. Companies are turning to strategic outsourcing to improve their competitive nature and enhance profitability (Marquis et al., 2015). Various studies have so far focused on the sustainability of firms from a theoretical perspective. A very few of these studies have focused on the application of sustainability principles to day-to-day business activities from the perspective of management (del Mar Alonso-Almeida et al., 2014; Huo et al., 2020). This research aims to provide a management perspective on the sustainability of firms and fills the gap in previous investigations. Organizational sustainability (OS) is among the approaches to sustainability that provide a management perspective to the organizations involved in the day-to-day operations of businesses (Morioka and Carvalho, 2016).

Incorporating this working practice with long-term goals has been a struggle for managers. Mangers of such organizations try to balance profitability and competitiveness through their management skills. Whereas, giant firms have been concentrating mainly on the development of operating systems (Sukitsch et al., 2015). There are certain ways through which organizations try to stay competitive. These include a search for sustainable practices and existing innovations in other markets (Falle et al., 2016). This can aid in better resource utilization and establish the circumstances for gaining a competitive advantage (Falle et al., 2016). The primary focus of OS is to ensure that current organizational demands would not jeopardize future generations' priorities. Various programs have been launched in this regard to promote sustainable consumerism and industrial practices (Keskin et al., 2013).

Sustainable practices of organizations differ from each other. These variations are based on the size of the company, level of commercial maturity, strategy development, and organizational structure. These sustainable practices involve the adoption, analysis, and assessment of potential adjustments for sustainable practices (Marquis et al., 2015). With these practices, organizations successfully implement their actions in the quick, intermediate, and long-term time frames. These practices enhance the creativity and resources of the organizations to strengthen sustainability. In addition to economic performance, environmental and social factors are also considered in the stakeholders' assessments. Sustainability reports are by far the most widely used and acknowledged globally. Such reports have risen in popularity among the various ways to document those activities (Thijssens et al., 2016). There is a recent consideration that the competitiveness of enterprises may be strengthened with the possible implementation of strategic human resource practices. There is an understanding that the human resource (HR) role has transitioned away from its conventional administrative position. It is now more focused on value creation and strategic role (Ulrich, 1997). An HR business partner framework was developed and applied first by US companies. It provides a conceptual perspective for the aforementioned organizational change. Around the year 2000, European companies began to restructure their HR departments to reflect the prototype HR business partner concept Szierbowski-Seibel and Kabst, 2018. According to the theoretical perspective, a fundamental feature of the HR business associate model is the rigid task separation inside the HR function (Szierbowski-Seibel and Kabst, 2018).

Statutory requirements are normally fulfilled by common administrative service centers which are separate from HR functions. Whereas in centers of excellence, complicated and knowledge-intensive activities are processed. The fastest-growing component of outsourcing is human resource outsourcing (HRO) which involves moving HR functions outside of an organization's boundaries (Martin, 2008). The strategic outsourcing focus on the commitment of operational performance, the transfer of specialized knowledge, higher control systems, and lesser risk. HR strategic outsourcing involves the strategic integration of practices which facilitate the switching of inward HR administration approaches toward an outward business approach (Glaister, 2014). The financial crisis has accelerated the impetus for HR outsourcing. HR outsourcing caused a fundamental shift in the norms of human resource function. It resulted in a cultural crisis of ideas, beliefs, and values (Zagelmeyer and Gollan, 2012). Therefore, HR function is now under pressure to change its rules and working methods (Zagelmeyer and Gollan, 2012). More precisely, strategic outsourcing of HR is now in practice. Generally, the HR function framework recommends the minimization of operational responsibilities assigned to the main HR function. It lays the foundation for HR function to become a strategic partner (Ulrich, 1997). Outsourcing of HR activities is a second management paradigm that European firms have acquired from US organizations (McCracken and McIvor, 2013). The outsourcing of HR activities and processes to a business organization is referred to as HRO.

HRO has increased in all dimensions of the HR function, according to studies published since 2000 McCracken and McIvor, 2013. A typical justification for HRO is based on the intention that HRO saves money. Moreover, research and practice imply that HRO has an impact on the HR function and is linked to the HR function's strategic integration (Szierbowski-Seibel and Kabst, 2018). Another approach for obtaining a competitive advantage for enterprises has been directed toward organizational capabilities. It is vital to understand why some organizations engage in sustainability strategy initiatives while others don't. It is even more important to know what activities and practices of enterprises help in dealing with the problems of sustainability (Schrettle et al., 2014). It is also worth noting that capability refers to the ability to learn, improve, and adapt to the challenges of competitiveness. Organizational capability (OC) differs from organizational capacity which refers to a more restricted holding, accommodating, or receiving ability (Rashid et al., 2015). Organizations must be able to adapt and employ new technology for environmental mitigation as well as other sustainability issues in order to accomplish long-term sustainability (Bhupendra and Sangle, 2015). Integrating multiple resources to adapt to external changes can be considered as an example of organizational capabilities developing an eco-based competitive edge. Some competencies such as organizational learning, connection development, common vision, interconnectivity, and technology sensing/response are critical in an environment-friendly sustainability approach (Amui et al., 2017). Therefore, due to its importance for achieving OS, OC was utilized as a mediator in the current investigation.

Among various strategies to opt for organizational sustainability in terms of economic, social, and environmental aspects, human resource management is seen as an emerging trend. A lot of research has been conducted on human resource management for organizational performance indicating that human capital could be utilized for keeping the competitive edge of organizations at par (Piwowar-Sulej, 2021). Nearly, all of the previous investigations have looked into the triple-down effects of HRM on OS accounting for social, economic, and environmental concerns. There has been a gap in finding the impact of HR strategic outsourcing on organizational sustainability (Piwowar-Sulej, 2021). This could also lead to a sustainable organization in order to maintain its competitiveness among other business organizations. To fill this gap, this study tries to find the possible association of HR strategic outsourcing with organizational sustainability.

Moreover, the proficiency of human resource practitioners is a multidimensional approach that can mediate the relationship of strategic human resource outsourcing with organizational sustainability. This kind of proficiency is administered through HR competency (Lo et al., 2015). Another critique of previous proficiency strategies is that they do not show how proficiencies are incorporated into effective organizational outcomes (Lo et al., 2015). Therefore, the authors try to find out its mediating role between HRO and OS. This study tries to answer some questions like possible associations between strategic HR outsourcing and OS, strategic HRO and organizational capabilities, and strategic HRO and HR proficiencies. This study also investigates the mediating effects of organizational capabilities and HR proficiencies between strategic HRO and organizational sustainability.

## Theoretical support and hypotheses development

Through his observations of dynamic economic systems, Coase (1937) created transaction cost theory (TCT). The TCT framework helps corporate leaders in determining the best cost-effective options among purchasing external services through an outsourcer and producing services and products through in-house sourcing. TCT arose as a startup paradigm, necessitating a transition in leadership interests from keeping possession of procedures for economic benefits and efficiencies to strategic outsourcing. The TCT framework is utilized in current research to investigate the study questions. Its theoretical model is frequently employed to assist managers in strategic decision-making that will lead to financial or increased efficiency (Coase, 1937).

Economic factors, incurring cost, and efficiency are the theory's main components. TCT's core premise is based on the assumption that administrators may export particular corporate tasks based on perceived immediate financial advantage before considering indirect costs. The purpose of outsourcing specific HR operations is to obtain advantageous operational costs or boost effectiveness by engaging outsourcing services to handle HR functions (Butler and Callahan, 2014). The resource based view (RBV) theory is also considered as an alternative theory to TCT and is frequently utilized by academics to investigate outsourcing. The RBV focuses on enhancing resources and competencies which are particular to a company and might even improve the company's competitive position (Sonfield, 2014). Researchers consider RBV as a secret sauce that provides a distinguishing advantage and leads to a sustainable competitive advantage. RBV is based on an organization's unique resources and capabilities. Throughout the framework of RBV, HRO gives an organization a competitive edge by creating innovative HR practices and developing resources that add value to the company (Boon et al., 2018). In the current study, strategic HR outsourcing provides a competitive edge for organizations by developing customized HR practices which are difficult to copy. Strategic HR outsourcing also tries to develop resources that add value to the company. Researchers are using the RBV theories to understand the outsourcing decisions of the company. These decisions focus on differentiated resources and advantage tactics. Moreover, the creators of RBV overlooked the associated cost, which is the fundamental driver of outsourcing services (Oduwusi, 2018).

An underlying principle of RBV focus on physical assets and human capital which are considered as key strategic resources for organizations (Oduwusi, 2018). Hence, the RBV offers a framework for developing resource strategies that would maximize organizational sustainable development. The RBV theories consider fundamental resources to be crucial to organizational performance. It refers to the idea of a company keeping control over key capabilities whilst outsourcing less valuable ones (Agburu et al., 2017). RBV theory, on the other hand, is not an appropriate paradigm for establishing core or critical capabilities for an organization (Liu et al., 2021). RBV and TCT provide fundamental support to the strategic human resource outsourcing in the current scope of investigation for organizational sustainability.

The RBV has an emphasis on the organization's internal capabilities and underlines the necessity for an organizational balance between exploitation of current resources and acquisition of new capabilities. Although according to Sandhu et al. (2018), HRO can be embedded inside a larger transformation program to help produce meaningful, unique, distinctive, and non-substitutable capabilities. However, the research doesn't really describe how this value might be driven through HRO involvement. Furthermore, distinguishing between core and peripheral HR operations is difficult. There is still a need to address the interaction between various HR processes (Wallo and Kock, 2018).

Various approaches have been adopted to combine RBV and TCT. These approaches consider combining of governance concerns with the financial intermediation associated with acquiring, safeguarding, and expanding resources efficiently (Brons, 2020). Whereas, these theories presume that HRO is a deliberate treatment with a well-articulated justification. This is rarely the case in actuality. It is equally important to think about the social as well as the financial factors which influence behavior (Glaister, 2014). Since HRO is a metaphorical approach to the outer environment, this is necessary to investigate whether HR rationalizes outsourcing with its own interests or amounts to the reasons due to which HRO becomes the active approach (Yingfei et al., 2021).

### Strategic HR outsourcing and organizational sustainability

Organizations are turning to outsourcing for many corporate tasks because of the promise of cost savings. Overall global revenue from HR outsourcing has surpassed $200 billion (Siew-Chen and Vinayan, 2016). Throughout difficult economic circumstances, corporate executives should employ specific measures to increase productivity. The HR information system, retraining, and employment are all general HR functions that are outsourced. A few of the critical objectives for outsourcing HR functions, according to Ohaegbu (2015), include (a) lowering expenses by trying to leverage third-party expertise and scale, (b) shifting HR focus to more key strategies, or (c) acquiring expertise and information insufficient in the respective organization (Hoang, 2018).

Since managers are utilizing outsourcing tactics as a strategy to respond to distinct organizational difficulties, the goal of HR outsourcing varies between each firm. HR outsourcing according to Letica (2014), entails a decision by business management to delegate specific non-core responsibilities to an external partner who may have the skills to do that activity more efficiently than in-house personnel. According to Ohaegbu (2015), outsourcing transaction services carries a lower risk of harm than engaging third parties to administer more strategic services, which may be better managed in-house. Because of the continuous interest of company executives in outsourcing techniques, many firms are looking at outsourcing various in-house tasks. Information technology outsourcing has been the focus of many related studies (Chang and de Búrca, 2016). HR outsourcing practices have recently piqued the interest of several corporate executives. Outsourcing might be prompted by market situations and developments (Sim and Kaliannan, 2016). During the financial crisis of 2007 and 2008, businesses tried a variety of cost-cutting measures such as reorganizing corporate units and off-shoring. HR outsourcing activity is expected to stay high in the future, owing to organizational leaders' continued use of strategies to achieve strategic goals and adjust to changing market conditions (Sim and Kaliannan, 2016). When a company works to grow its financial returns, it proactively analyses and controls its economic effects on internal and external stakeholders. It implements regulations to ensure that natural resources do not get deteriorated to the point that future generations would be harmed by environmental degradation (Dwivedi et al., 2021).

According to the RBV model, human resource outsourcing is critical to achieving sustainability (Sonfield, 2014). According to the approach, firms can gain a long-term strategic advantage in a challenging environment by honing a variety of capabilities. Employees can benefit from HRO methods and practices in structuring and guiding their perspective and behavior toward a sustainable future (Shibin et al., 2020). Human resource outsourcing aids in the following areas: identifying and framing key indicators for employees that contribute to sustainability; bringing internal and external sustainability training for staff; empowering knowledge sharing and strategic planning programs within the organization related to business, society, and environmental sustainability; and rewarding and recognizing individuals' ideas and accomplishments to achieve the organization's sustainable necessities (Shibin et al., 2020).

In past, some scholars have tried to identify the possible relationships of different HR practices for the sustainable development of business organizations and found significant associations, such as in Dwivedi et al. (2021). Similarly, some of the researchers e.g., Macke and Genari (2019) suggested that different human resource practices pave the path for achieving sustainable development of business organizations. Organizational sustainability has been a significant target for a lot of researchers in the recent past and therefore, HR related researchers have got deep understanding of this phenomenon for environmental, social and economic sustainability of the organizations. Strategic human resource outsourcing is one of the HR management practices. So, keeping in view the importance of HRM in organizational sustainability and recommendation of related literature, author proposed the following hypothesis.

**H**_**1**_**:** Strategic HR outsourcing may lead to organizational sustainability.

### Organizational capabilities

According to Grant (1991), organizational capabilities refer to a company's ability to deploy its resources (both real and intellectual) to complete a task to improve its performance. Organizational capabilities in this study are comprised of three elements: strategic marketing capability, diverse stakeholder relationship capability, and efficiency of the operations. Organizational capabilities which receive a huge amount of attention improve the impact on organizational efficiency and financial performance. Moreover, an organization's internal strengths or organizational skills define how it gains an advantage over competitors with the final target being greater performance (Shurafa and Mohamed, 2016).

The company's key role in ensuring continuous improvement is called capability. Moreover, according to some studies, an organization's capability is a valuable commodity, unique knowledge, exceptional commodity, and strategic asset (Hindasah and Nuryakin, 2020). Researchers and scholars believe that the capability and distinctive capacity of a successful firm determines its competitive advantage. Organizational capability can be defined as an organization's ability to manage its company by having the elements of sensing to address the changing environments rapidly, decrease operational activities, and adjust to environmental and market globalization changes (Hindasah and Nuryakin, 2020).

According to Freeze and Kulkarni (2007), organizational capability is linked to concrete knowledge assets. The findings of this study revealed the importance of organizational responsibility in utilizing the processes and technology provided by the organization as a framework for developing a personally liable human resource to support the knowledge in an organization. OC also gives an insight into the equipment and procedures required to effectively improve the organization's knowledge ability. This study also discovered that five managerial capabilities (skills, acquisition, procedures and policies, statistics, and information documentation) had a strong link with the particular sequence in the organization's business (Freeze and Kulkarni, 2007).

Another research indicating the role of organizational capabilities stated that SMEs' businessmen must bear responsibility for the organization's staff's survival and success (Ong et al., 2010). Furthermore, the unpredictable conditions will have an impact on the operation's flexibility and the ability of enterprises to respond to changes. The findings of the study revealed that innovations have a significant role in determining SMEs' competitive advantage and productivity. Some scholars claimed that SMEs were not given enough attention to establish a successful strategy (Hindasah and Nuryakin, 2020). Due to a major shortage of resources and a limited ability to innovate, SMEs confront numerous challenges. Business organizations must have organizational capabilities, organizational strategy, and top performance accomplishments to sustain their competitiveness (Hindasah and Nuryakin, 2020).

External and internal network capability on company performance were investigated by Lee et al. (2001). The direction of entrepreneurship, technological capability, and committed financial resources were used to implement internal capability. The findings of the study revealed that internal ability has an impact on innovative performance (Lee et al., 2001). Inner ability and a cooperative relationship have a big impact on a company's performance. Similarly, Lawson-Body and O'Keefe (2006) demonstrated that organizational capability based on knowledge systems can provide strategic benefits to corporate organizations through customer loyalty. The findings of this study also revealed that online commerce has an impact on corporate companies' multi-organizational relationships with their devoted clients.

Carvalho and dos Reis (2012) explored the influence of organizational capabilities *via* IT capability in analyzing the management's perspective on the implementation of innovative techniques and the company's relationship. (Carvalho and dos Reis, 2012) indicated that enterprises used information technology to successfully place the creative product in the market. According to the findings of the study, an organization contributes to the development of an idea and commercialization of new products. All the supporting literature available on organizational capabilities indicated that OC have the potential to influence the organizational performance and achieve sustainability for the organizations. It was also assumed that through strategic human resource outsourcing, organizational capabilities could be improved. Therefore, authors decided to explore the following role of OC in current research.

**H**_**2a**_**:** Strategic HR outsourcing has an influence on organizational capabilities.

**H**_**2b**_**:** Organizational capabilities play a role in organizational sustainability.

**H**_**4**_**:** Organizational capabilities mediate the relationship between strategic HR outsourcing and organizational sustainability.

### HR proficiency

A few researchers determined that non-core HR tasks and activities should be outsourced, while others found that core HR functions and activities should be preserved (Susomrith and Brown, 2013). HR departments can focus on higher-value operations by outsourcing non-core functions, which has been shown to increase an organization's performance. To ensure long-term success, a company should focus on its core skills while eliminating non-core operations that consume resources but add little value (Susomrith and Brown, 2013). The problem emerges while deciding about which HR functions must be outsourced and what should be kept in-house because there is no easy way to distinguish between the two. Due to the low number of empirical studies, Cooke et al. (2005) found from their investigation that it is difficult to make a firm conclusion on the sorts of HR functions that should be outsourced.

According to Patel et al. (2019), operations are suited to outsourcing if they are routine, properly delineated, measurable, managed at arm's length, rapidly supplied by experienced suppliers, and supplied in a competitive environment. In addition, Mishra et al. (2018) identified six characteristics that a corporation must examine while outsourcing. Dependence concerns, contagion consequences, confidence, comparative proficiency, strategic capabilities, and dedication vs. flexibility are all examples of these. Few researchers believe that the potential of HR to add a competitive edge is a factor in deciding to choose whether or not to outsource HR operations (Papageorgiou, 2018). The proficiency required of HR professionals is totally different from all those previously identified with HR.

Papageorgiou (2018) argue that HR should build vendor management, technology, law, and finance abilities, and these skills should be based on the performance of a product whenever possible. According to them, HR will rely less on administrative skills and more on centers of expertise, senior strategic HR analysts, and system designers. Hays (2019) identified a set of behavioral HR proficiencies including identity and elevated effective communication, constructing customs, effectiveness, performance, and entrepreneurship. HR proficiency helps in introducing and overcoming challenges, analyzing company strategy and co-producing strategic plans, participating in skill development, leadership, and participating in talent planning and organizational design through technology acquisition (Hays, 2019).

According to comments from practitioners, HR is still struggling to develop strategic management proficiency in areas like costing and analysis of data, program and project management, budgeting acumen, and cross-functional competence (Vrontis et al., 2022). HR practitioners are increasingly concerned about the profession's expanding skills gap and the challenge of poor business expertise. Instead of the new HR proficiency of leadership and internal consulting, they focus their development operations on inward-facing personal proficiencies. Taponen and Kauppi (2020) believes that HRO activity is based on HR departments' failure to enhance their own procedures internally. HR departments prefer to place a premium on service efficiency over strategic external pressures (Patel et al., 2019). The former is provided by HRO due to the immediacy of required goals, but responding to these constraints necessitates a fresh range of HR proficiencies (Patel et al., 2019). The suggested literature in the field of strategic HR outsourcing has focused on several practices which could lead to better organizational performances. Having a focus on HR proficiency in terms of competency could lead to better organizational performances. Proficient HR capital might be utilized as a useful strategic tool of HR outsourcing. Therefore, keeping in view the significance of HR proficiency in the field of HR management, author developed the following hypothesis.

**H**_**3a**_**:** Strategic HR outsourcing has an influence on HR proficiency.

**H**_**3b**_**:** HR proficiency plays a role in achieving organizational sustainability.

**H**_**5**_**:** HR proficiency mediates the relationship between strategic HR outsourcing and organizational sustainability.

**Figure 1:**
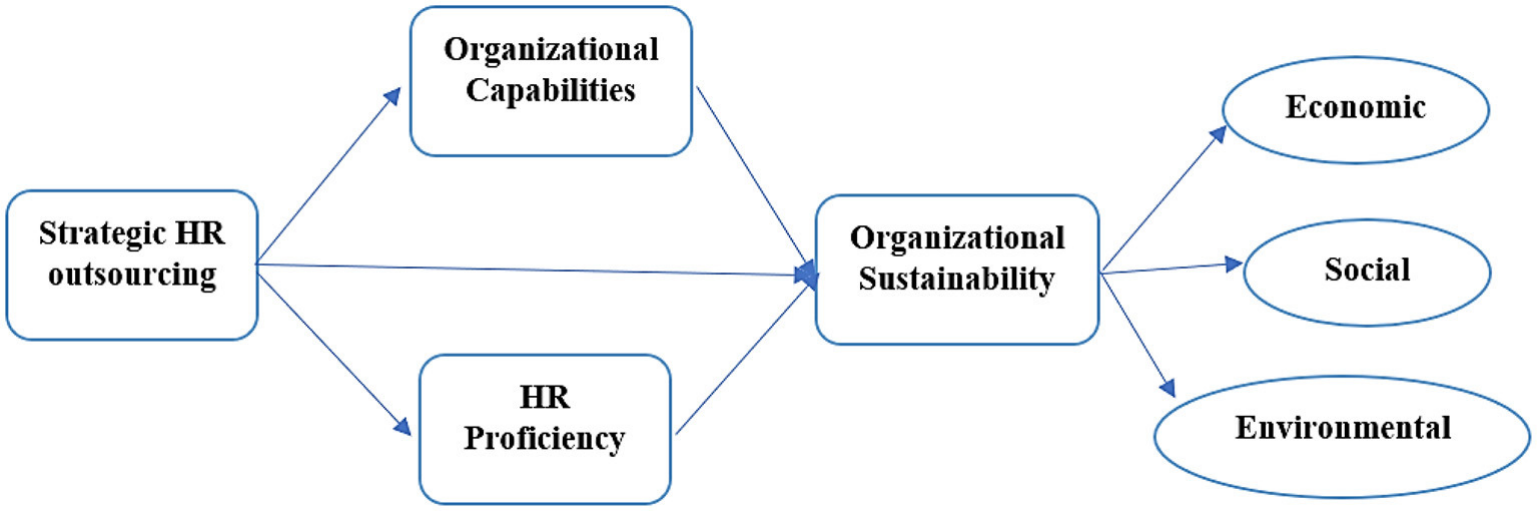
Theoretical framework.

This research is based on the following framework given below (see Figure 1).

## Methodology

The current study used a quantitative method with a deductive approach, in which hypotheses were developed and tested to discover how specific factors affected other variables. Researchers utilize this practice to ensure that there is no bias. To collect data for this quantitative study's analysis, self-administered surveys were used. Surveys provide a high level of general capability in representing a large population. Moreover, they are useful for convenient data collection, have good statistical significance, little observer subjectivity, and provide precise results (Nawaz et al., 2022). The study's target group was China's HR professionals working in business industry. In this study, the convenience sample approach was employed to obtain data from respondents *via* physically distributed questionnaires. The data collection process took 2–3 months to complete. The total number of questionnaires distributed was approximately 500, and we obtained 425 responses, yielding an 80% response rate; 400 of those 425 responses were viable and used for data analysis. Because the data collection technique was initially delayed, reminders were sent to selected respondents to expedite the process. Our study unit of analysis was HR professionals.

### Statistical tool

In this study, the Smart-PLS 3 was utilized to evaluate the data. In our study, structural equation modeling was used. Partial Least Square is frequently utilized in management and social sciences since it is a variance-based structural equation modeling technique (Avotra et al., 2021). PLS-SEM is also a causal modeling technique whose purpose is to increase the explained variance of latent dependent components. Researchers regard PLS-SEM as a “magic bullet” for dealing with empirical findings with a limited sample size (Hair et al., 2011). Smart-PLS is easy to use and contains a wealth of complex capabilities (Garson, 2016). Furthermore, the Smart-PLS approach is best suited to complex equation studies (Nawaz et al., 2019; Hao et al., 2020). This study follows the suggestions of Wong (2013) to properly calculate the values of beta, reliability, and standard error and ensures that all of those indicators are part of their respective latent variables with outer loadings of 0.7 in the reflecting outer model evaluation.

### Measurement

In this study, a five-point Likert scale ranging from strongly agreed to strongly disagree was employed to capture respondents' responses. When assessing the dependability of each variable, the Cronbach alpha value should be larger than 0.7. (Hair et al., 2019).

#### Strategic HR outsourcing

This study utilized the scale of Sim et al. (2021) which consists of 14 items. The value of Cronbach's alpha is 0.936, which is acceptable as compared to the benchmark value.

#### Organizational capabilities

Organizational capabilities were measured by utilizing the 14 items scale of López-Cabarcos et al. (2015). The value of Cronbach's alpha is 0.941, which is well above the required value.

#### HR proficiency

In this study HR proficiency was measured by adapting the scale of Yamao and Sekiguchi (2015) which consists of five items. The Cronbach's alpha value of HR proficiency is 0.876, which is acceptable as compared to the benchmark value.

#### Organizational sustainability

This adopted the 11 items of the scale of Jung et al. (2021). The Cronbach's alpha is 0.931 for economic sustainability, 0.906 for environmental sustainability, and 0.876 for social sustainability, which is well above the benchmark value.

### Demographic details

Table 1 shows the demographic characteristics of the survey respondents. Demographic variables describe the study sample and determine if samples are representatives of population interest. In our study demographic variables, gender and education are used to determine the impact of strategic outsourcing on the basis of gender and respondent's education level. The study had 400 persons in all, with 221 men and 169 women participating. Bachelor's degree holders made up 59% of all participants, while master's degree holders made up 41%. Respondents were chosen using the convenience sampling technique to obtain data in a cost-effective and time-saving manner.

**Table 1:** Demographic analysis.

**Variable**	**Groups**	**No of respondents**	**Total**
Gender	Male	221	400
	Female	169	
Education	Graduate	260	
	Masters	140	400

### Common method bias

Table 2 shows the overall variation explained by single-factor analysis for each of the variables tested. It highlights the common technique bias, as well as the bias of the questionnaire. The percentage of variance for one item must be less than 50% (Yong and Pearce, 2013). There is no bias in the data because the total variation described in this study is less than 50%.

**Table 2:** Common method biasness.

**Component**	**Initial Eigenvalues**			**Extraction Sums of Squared Loadings**		
	**Total**	**% of Variance**	**Cumulative %**	**Total**	**% of Variance**	**Cumulative %**
1	14.659	32.576	32.576	14.659	32.576	32.576
2	4.503	10.007	42.583			
3	2.854	6.342	48.925			
4	2.4	5.334	54.259			
5	1.931	4.29	58.549			
6	1.739	3.864	62.413			
7	1.482	3.294	65.707			
8	1.372	3.05	68.757			
9	1.271	2.824	71.581			
10	1.173	2.608	74.189			
11	1.029	2.287	76.475			
12	0.914	2.032	78.507			
13	0.886	1.97	80.477			
14	0.841	1.87	82.347			
15	0.773	1.718	84.065			
16	0.711	1.58	85.645			
17	0.656	1.459	87.104			
18	0.548	1.217	88.321			
19	0.546	1.213	89.533			
20	0.488	1.083	90.617			
21	0.464	1.032	91.648			
22	0.43	0.955	92.603			
23	0.404	0.899	93.502			
24	0.354	0.786	94.288			
25	0.289	0.642	94.93			
26	0.277	0.616	95.546			
27	0.269	0.597	96.143			
28	0.218	0.485	96.628			
29	0.207	0.459	97.088			
30	0.205	0.455	97.543			
31	0.171	0.381	97.924			
32	0.15	0.334	98.258			
33	0.145	0.323	98.58			
34	0.117	0.26	98.84			
35	0.098	0.218	99.059			
36	0.084	0.186	99.245			
37	0.07	0.157	99.402			
38	0.058	0.128	99.53			
39	0.049	0.109	99.639			
40	0.041	0.09	99.729			
41	0.037	0.083	99.812			
42	0.032	0.07	99.883			
43	0.024	0.054	99.937			
44	0.021	0.046	99.982			
45	0.008	0.018	100			

## Data analysis

### Measurement model

**Figure 2:**
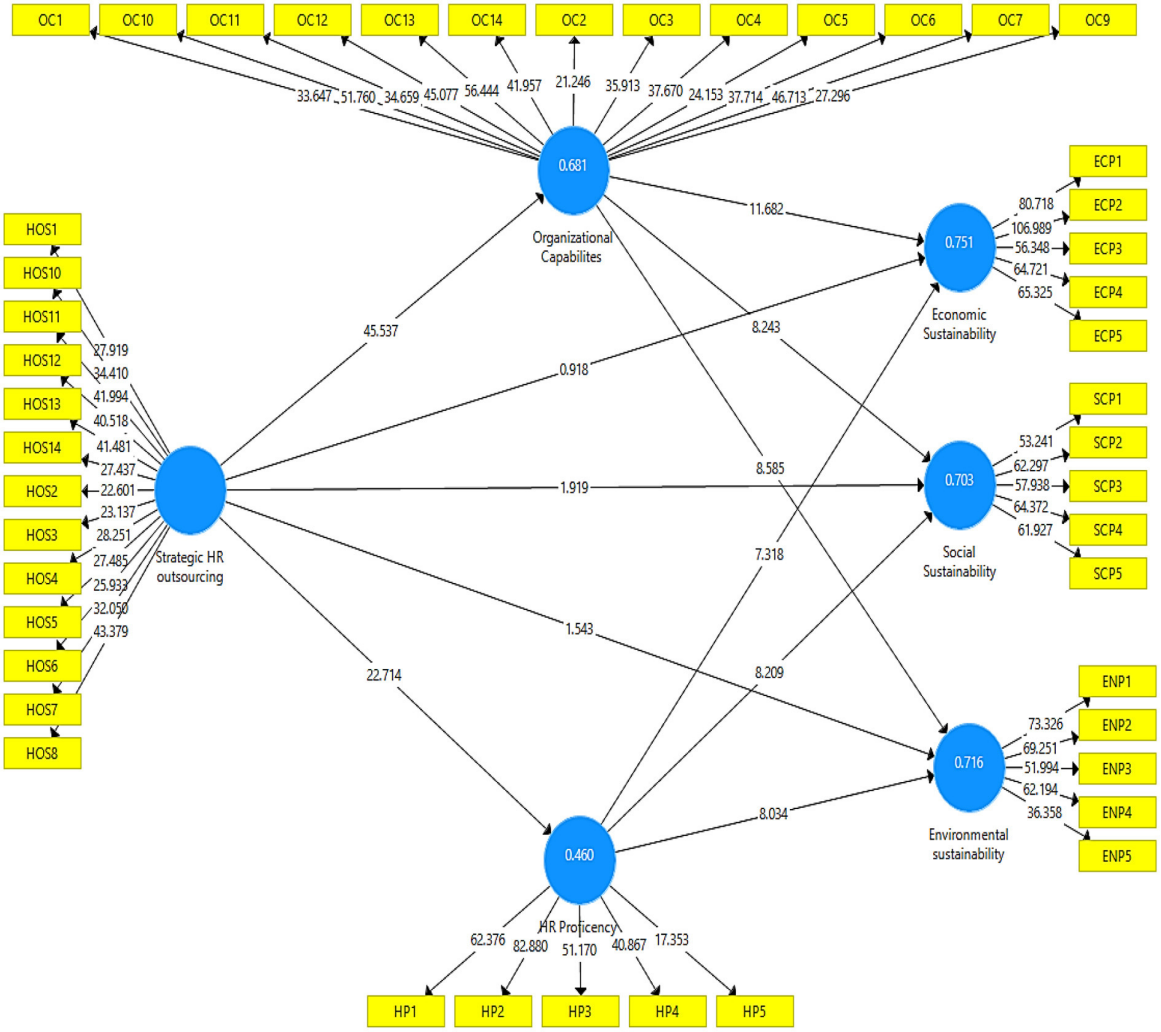
Output of measurement model algorithm.

The output measurement model's algorithm is represented in Figure 2. This diagram depicts the effect of independent variables on the dependent variables of the study.

Table 3 illustrates the factor loadings for each research construct, which are strategic HR outsourcing, organizational capabilities, HR proficiency, and organizational sustainability. In addition to the VIF values, the table shows the extracted composite reliability and average variance (AVE). The factor loading describes an item's contribution to the variable and must be greater than 0.60 (Jordan and Spiess, 2019). All of the factor loadings in this study are more than 0.60, indicating that the factor loadings are fair. The model's collinearity issues are validated by the variation inflation factor (VIF). The current study's outer VIF result is also less than 5 (range from 1.804 to 4.211), showing that the model is not collinear. In addition, the present inner VIF result is less than 5 (between 1.508 to 2.475). The AVE values in Table 3 are larger than 0.60, showing the presence of convergent validity. The composite reliability was better than 0.70, putting it in the highly satisfactory category (Peterson and Kim, 2013).

**Table 3:** Factor loadings cronbach alpha, composite reliability, and AVE.

**Constructs**	**Items**	**Loadings**	**Alpha**	**rho_A**	**CR**	**AVE**
Economic sustainability			0.931	0.931	0.948	0.784
	ECP1	0.895				
	ECP2	0.91				
	ECP3	0.868				
	ECP4	0.882				
	ECP5	0.871				
Environmental sustainability			0.906	0.909	0.93	0.728
	ENP1	0.873				
	ENP2	0.875				
	ENP3	0.834				
	ENP4	0.871				
	ENP5	0.811				
Social sustainability			0.876	0.885	0.91	0.671
	SCP1	0.85				
	SCP2	0.874				
	SCP3	0.87				
	SCP4	0.87				
	SCP5	0.858				
Strategic HR outsourcing			0.936	0.939	0.944	0.563
	HOS1	0.756				
	HOS2	0.739				
	HOS3	0.726				
	HOS4	0.751				
	HOS5	0.742				
	HOS6	0.719				
	HOS7	0.721				
	HOS8	0.761				
	HOS10	0.758				
	HOS11	0.79				
	HOS12	0.795				
	HOS13	0.774				
	HOS14	0.721				
HR proficiency			0.876	0.885	0.91	0.671
	HP1	0.862				
	HP2	0.88				
	HP3	0.841				
	HP4	0.803				
	HP5	0.698				
Organizational capabilities			0.941	0.943	0.948	0.587
	OC1	0.747				
	OC2	0.658				
	OC3	0.748				
	OC4	0.768				
	OC5	0.698				
	OC6	0.772				
	OC7	0.799				
	OC9	0.761				
	OC10	0.808				
	OC11	0.764				
	OC12	0.802				
	OC13	0.833				
	OC14	0.782				

To examine discriminant validity, the HTMT ratio and the Fornell and Larker Criteria were utilized (see Tables 4, 5). These tests examine whether the difference exists or not between the variables. The HTMT ratio should be smaller than 0.90 to ensure the discriminant validity of a variable (Yong and Pearce, 2013). The current study's HTMT ratio was smaller than 0.90, showing that discriminant validity existed. According to the Fornell and Larker Criteria, the value at the top of the column must be greater than the value below that column (Henseler et al., 2015).

**Table 4:** Discriminant validity (HTMT ratio).

**Constructs**	**EcS**	**EnS**	**HP**	**OC**	**SocS**	**SHRO**
Economic Sustainability						
Environmental sustainability	0.963					
HR Proficiency	0.859	0.866				
Organizational Capabilities	0.797	0.824	0.846			
Social Sustainability	0.748	0.792	0.790	0.856		
Strategic HR outsourcing	0.717	0.76	0.731	0.767	0.747	

**Table 5:** Discriminant validity (fronell and larcker criteria).

**Constructs**	**EcS**	**EnS**	**HP**	**OC**	**SocS**	**SHRO**
Economic sustainability	**0.885**					
Environmental sustainability	0.81	**0.853**				
HR proficiency	0.778	0.775	**0.819**			
Organizational capabilities	0.843	0.812	0.773	**0.825**		
Social sustainability	0.786	0.814	0.771	0.802	**0.864**	
Strategic HR outsourcing	0.722	0.712	0.678	0.776	0.708	**0.751**

*Bold value shows the significance between variable*.

A R-Square score of 0.50 or higher implies that the model is substantial and good (Archer et al., 2021). The R-square values for the variables in the current study are close to or more than 0.50, indicating that the model is adequate. As measured by q-square, cross-validated redundancy should be greater than zero (Henseler et al., 2015). The Q-square values for the variables in the current study are more than zero, indicating that the model is significant (see Table 6).

**Table 6:** R-Square values and Q-Square values for the variables.

	**R2**	**Q2**
HR Proficiency	0.459	0.21
Organizational Capabilities	0.682	0.105
Organizational sustainability	0.818	0.145

### Structural model

**Figure 3:**
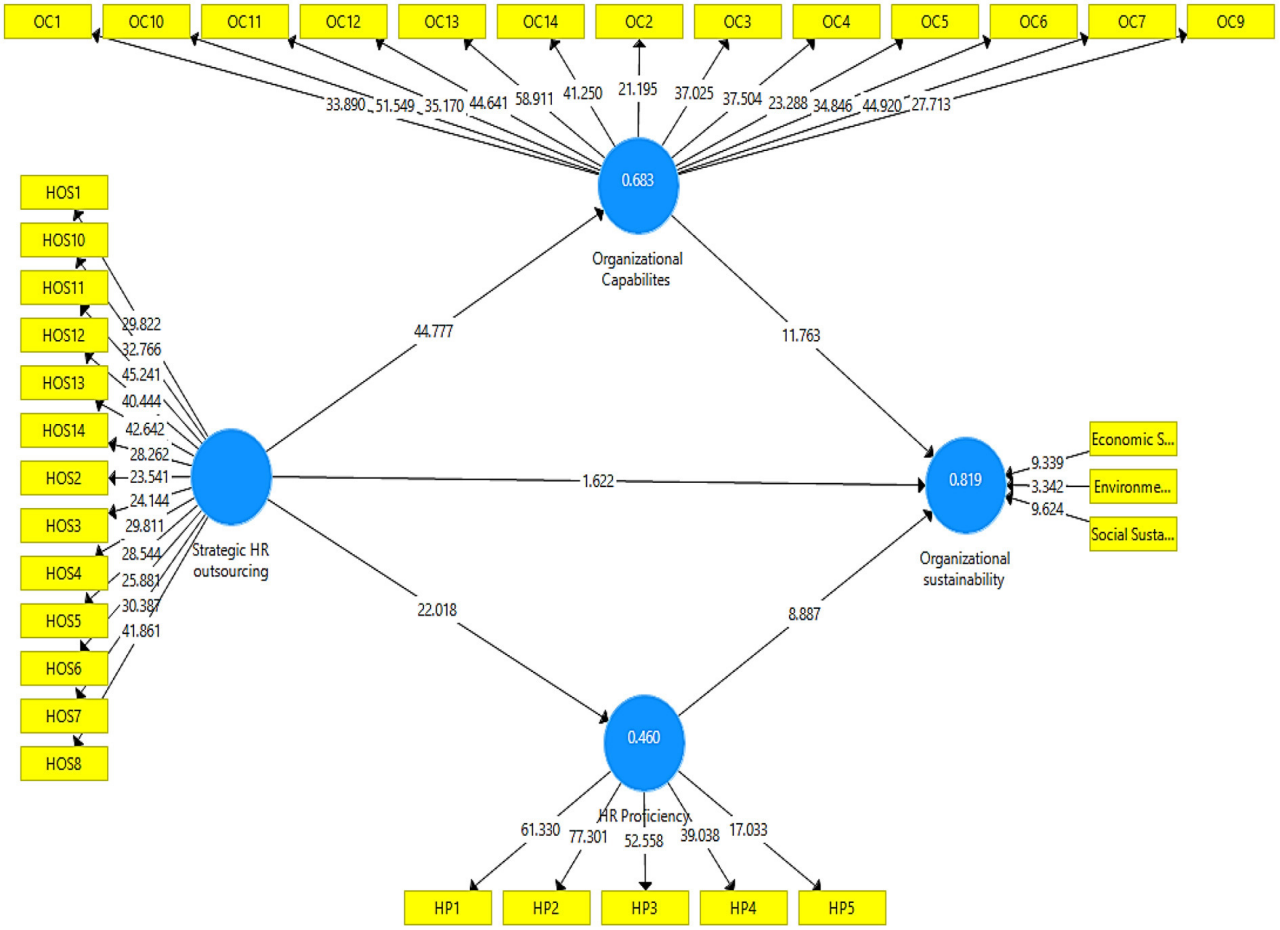
Output of structural model algorithm.

Following the successful analysis of the measurement model (Figure 3), which included the establishment of constructs as well as indicator reliability and validity, the structural model was evaluated to determine the coefficient of determination (R2), the significance of the path coefficient, and the relevance of the path coefficient.

R2 explains the probability in endogenous variables that occurred as a result of an exogenous variable. R2 should be greater than 0.10 if possible. R2 value criteria provided by Hair et al. (2013) include 0.75, 0.50, or 0.25 for endogenous latent variables, which can be defined as considerable, moderate, or weak as a general rule of thumb. An R2 value of HR proficiency is 0.459, which means 45% variation in HR proficiency happens due to all exogenous variables. Similarly, the R2 value of organizational capabilities is 0.682 and their organizational sustainability is 0.818. The hypothesis testing was carried out using a bootstrapping technique, with a resample of the number of 5,000 bootstraps.

The results of H1 shows that strategic outsourcing has insignificant and positive impacts on organizational sustainability (β = 0.076, *p* = 0.105). As value of P is greater than 0.05, H1 was rejected. The H2 findings shows that strategic HR outsourcing has a positive and significant impacts on organizational sustainability (β = 0.826, *p* = 0.000). Hence H2 is accepted. Similarly, the finding of H2b shows that organizational capabilities has a significant and positive impact on organizational sustainability (β = 0.530, *p* = 0.000). The results of H3a shows that strategic HR outsourcing has insignificant impacts on HR proficiency (β = 0.678 *p* = 0). Hence H3a is accepted (see Table 7). The results of H3b demonstrate that HR proficiency has a significant and positive impact on organizational sustainability (β = 0.363, *p* = 0.000).

**Table 7:** Direct effects.

**Hypotheses**	**Relationship**	**Beta**	**SD**	**T value**	**P Value**	**Confidence interval**		**Decision**
						**LL**	**UL**	
H1	Strategic HR outsourcing -> Organizational sustainability	0.076	0.047	1.622	0.105	−0.015	0.164	Unsupported
H2a	Strategic HR outsourcing -> Organizational capabilities	0.826	0.018	44.777	0.000	0.790	0.862	Supported
H2b	Organizational capabilities -> Organizational sustainability	0.530	0.045	11.763	0.000	0.444	0.616	Supported
H3a	Strategic HR outsourcing -> HR proficiency	0.678	0.031	22.018	0.000	0.622	0.743	Supported
H3b	HR proficiency -> Organizational sustainability	0.363	0.041	8.887	0.000	0.292	0.443	Supported

This study adopts Preacher et al. (2016) method for performing the mediation analysis. The mediating role of organizational capabilities and HR proficiency were assessed between the relationship between strategic HR outsourcing and organizational sustainability. H4 proposes that organizational capabilities mediate the relationship between strategic HR outsourcing and organizational sustainability. Results show that the direct effect between strategic HR outsourcing and organizational sustainability was insignificant. However, the mediation effect of OC between strategic HR outsourcing and organizational sustainability was significant (β = 0.438, *p* = 0.000) so this shows that fully mediation effect of organizational capabilities between the relationship of strategic HR outsourcing and organizational sustainability (see Table 8). Similarly, an HR proficiency fully mediates the relationship of strategic HR outsourcing and organizational sustainability (β = 0.246, *p* = 0.000).

**Table 8:** Mediation effect.

**Hypotheses**	**Constructs**	**Total effect**		**Direct Effect**		**Indirect Effect**	
H4	SHO->OC->OS	0.76	0.000	0.076	0.105	0.438	0.000
H5	SHO->HP->OS					0.246	0.000

## Discussion

This research tried to focus on the strategic dimension of human resource management, specifically the outsourcing of human capital to other organizations in dealing. For achieving sustainable development of any corporate organization, it is necessary to work on the human capital of organizations. The current research looked at the possible relationships of strategic HR outsourcing with the sustainability of the organizations. It also looked into the direct relationships of strategic HR outsourcing with organizational capabilities and human resource proficiencies. This research also looked into the direct associations of organizational capabilities with organizational sustainability and human resource proficiencies with organizational sustainability.

Moreover, this research also focused on finding the mediating roles of organizational capabilities and the human resource proficiencies between strategic human resource outsourcing and organizational sustainability. The results indicated that strategic outsourcing of human resources could not develop an association with organizational sustainability. This indicated that there should be some indirect ways to achieve organizational sustainability while taking into account the human resource management practices. Only strategic human resource outsourcing is not enough to develop a sustainable organization. This is due to the fact that sustainable organizations are achieved through a mixed approach of different aspects among which HRM is one of the influencers.

This initiated a major scope for strategic HR outsourcing in improving the performance of organizations. Previously, some researchers tried to find out the impact of different human resource management practices on organizational performance and attained significant results, such as Dwivedi et al. (2021). Moreover, some researchers e.g., Macke and Genari (2019) also predicted that HR outsourcing could take part in sustainability practices of the organizations. Secondly, this research evaluated the possible associations between strategic human resource outsourcing and organizational capabilities. This indicated a strong association revealing the proper strategic HR outsourcing could play a significant role in enhancing capabilities of the organizations.

It is previously confirmed that corporations involved in business activities, are marginal for contemporary business organizations. For this purpose, these firms need strategic planning and certain organizational capabilities which help them achieve high returns and improved performance (Hindasah and Nuryakin, 2020). Therefore, strategic human resource outsourcing could help in developing organizational capabilities which in turn help in achieving sustainable performance. The results of the direct association of organizational capabilities with organizational sustainability were also in accordance with this notion and proved that proper organizational capabilities could help in achieving organizational sustainability. These results add to the literature on organizational management by confirming the results of Carvalho and dos Reis (2012) who indicated that enterprises used information technology to successfully place creative products in the market. This is an indication that improving the technological capabilities of a firm may add to the sustainable performance of firms.

Moreover, this study also found the significant role of HR strategic outsourcing in providing HR proficiencies to the organization. This indicated that proper outsourcing strategic practices of human resources to the companies involved help in improving the competency of the human capital of all organizations. It was emphasized by the HR professionals that the long-term success of the organizations could be achieved through developing and polishing the core skills of the professionals while reducing the cost of the resources which add little value to the organizational performance and consume more (Susomrith and Brown, 2013). The results of Susomrith and Brown (2013) provided an insight into the role of HR outsourcing in developing the HR proficiency of individuals involved in the organization. In accordance with these results, current research also proved that HR outsourcing may improve the HR proficiency of employees. Similarly, the direct association of HR proficiency was also analyzed in this study on achieving organizational sustainability. The results proved that proficient human capital could add its input to achieving sustainable development of the organization. These results are an indication that people with high proficiency contribute to the sustainable performance of the enterprises. These results also confirm the prospective role of HR proficiency toward sustainability by Yamao and Sekiguchi (2015).

This study also evaluated the indirect effects of organizational capabilities and the HR proficiency between strategic HR outsourcing and organizational sustainability. The results indicated that a direct relationship between the two could be shaped into a valuable association with the help of organizational capabilities and HR proficiencies of the employees. The results revealed that both of the mediators helped in achieving organizational sustainability through strategic human resource outsourcing. The results are supported by the findings of Taponen and Kauppi (2020) who hinted at the role of human resource strategic outsourcing in acquiring organizational sustainability through organizational capabilities. The results also got support from the findings of Patel et al. (2019) who emphasized the role of HR proficiencies in achieving organizational improved performance contributing to the sustainability of the organizations.

### Theoretical implications

The findings of this investigation have important theoretical consequences. This research adds significantly to the TCT and RBV theories. The cost of strategic outsourcing adds to the sustainable performance of organizations in a mediated way. Therefore, it has a contributory effect on TCT. Meanwhile, organizational capabilities indirectly contribute to the sustainable performance of organizations. Hence, it has implications for RBV theory. RBV theories are strengthened by the fact that capabilities are the resources of firms that add to the sustainability of organizations. The study first looked at the impact of strategic human resource outsourcing on long-term organizational viability. Because such a model has never been researched and explored previously, the current study significantly contributed to the existing literature on human resource management and organizational management.

Strategic human resource outsourcing was also found to play a significant effect in shaping organizational capacities and human resource proficiency, according to the study. By looking at organizational capacities and human resource proficiency as mediators, this study has added to the literature. The reader can see how important organizational competencies are in supporting the relationship between strategic human resource outsourcing and long-term organizational viability. It would also add to the research on the importance of human resource proficiency as a mediator between strategic human resources and tactical human resources. It would also contribute to the literature by highlighting the importance of human resource expertise as a mediator between strategic human resource outsourcing and organizational sustainability. This study will broaden readers' understanding of the resource based view of businesses while also emphasizing the importance of human resource outsourcing in the theory.

### Managerial implications

This research has some important practical consequences for company management. Human resource management strategies such as human resource strategic outsourcing, organizational competencies, and human resource proficiency, according to the findings of the study, are essential variables in building organizational sustainability in corporate businesses. As a result, the management of such companies should encourage their staff to interact efficiently in order to improve outsourcing procedures among competitors. It would be advantageous to hire supportive leaders who can allow smooth outsourcing activities between the firms. Another approach to optimize human resource outsourcing operations is to have a centralized system in place within the company.

Moreover, organizational capabilities and human resource proficiency are important indicators that help to maximize organizational sustainability. organizational capabilities of the employees could be enhanced through different approaches including training of the employees. moreover, their proficiency can also be achieved through training and could be evaluated through different competency analyses. As indicated by the results, it was also evident that proper human resource strategic outsourcing could also help in shaping the proficiencies and the capabilities of the employees of the organizations.

### Limitations and future directions

Although the current study was meant to evaluate Chinese HR experts, other professionals from other corporate businesses could be examined in future studies. As the study's environment is adjusted in future studies, the results for Western areas and other Asian countries (aside from China) may shift. Furthermore, a larger sample size would aid in the generalization of the data. A hybrid method can be used in future studies to gain a better understanding of the relationship between study factors. With the mediation of organizational capacities and human resource proficiency, the current study looked at the impact of one human resource management practice, human resource strategic outsourcing, on organizational sustainability. Future studies can examine the role of other human resource management practices such as compensation and benefits, employee retention, and coaching and development on organizational sustainability with mediating support of human resource competency.

## Conclusion

The necessity of the hour is to promote organizational sustainability through smart human resource outsourcing. HR outsourcing is the key contributor in the sustainable performance of enterprises as it adds to the human capital of organizations. The organizations get support from HR professionals of other organizations. This adds to the competitiveness of organizations. In the field of HR management, strategic outsourcing is proving its worth as a contributor to sustainable performance in current times. There has been a need to explore the roles of organizational capabilities and HR proficiency of employees toward organizational performance which lead to sustainability. Moreover, organizations are looking for strategies to improve organizational sustainability through various human resource management practices in this area. In this regard, the current study used specialists from several Chinese HR organizations to explore the impact of strategic human resource outsourcing on organizational sustainability. This approach proved to be helpful in the field of organizational management as it provides significant relationships of different aspects of HR management.

In order to achieve organizational sustainability, the study looked at the mediating impacts of organizational capacities and human resource proficiency. The study's findings revealed that strategic human resource outsourcing could not justify its benefit only in terms of organizational sustainability. While the findings revealed that strategic human resource outsourcing could be made more effective by combining both mediators, i.e., organizational capabilities and human resource proficiency, the findings also revealed that strategic human resource outsourcing could be made more effective by combining both mediators, i.e., organizational capabilities and human resource proficiency. This study also looked into the direct impacts of human resource strategic outsourcing practice on organizational capabilities and human resource proficiency. It proved that proper strategic human resource outsourcing practice could develop organizational capabilities and proficiencies among the employees. This also proved that organizational capabilities and human resource proficiency are important in developing organizational sustainability.

## Data availability statement

The original contributions presented in the study are included in the article/supplementary material, further inquiries can be directed to the corresponding author.

## Ethics statement

The studies involving human participants were reviewed and approved by the Shanghai University of finance and economics, China. The patients/participants provided their written informed consent to participate in this study. The study was conducted in accordance with the Declaration of Helsinki.

## Author contributions

LS conceived and designed the concept, collected the data, and wrote the article. The author read and agreed to the published version of the manuscript.

## Funding

This work was supported by the graduate innovation fund Project of Shanghai University of Finance and economics a Political Economic Study on the Downward trend and actual change of profit margin (CXJJ-2021-377). School-level Scientific Research Project of Anhui Polytechnic University Construction of High Quality Development index of Regional integration in the Yangtze River Delta (Xjky05201910).

## Conflict of interest

The author declares that the research was conducted in the absence of any commercial or financial relationships that could be construed as a potential conflict of interest.

## Publisher's note

All claims expressed in this article are solely those of the authors and do not necessarily represent those of their affiliated organizations, or those of the publisher, the editors and the reviewers. Any product that may be evaluated in this article, or claim that may be made by its manufacturer, is not guaranteed or endorsed by the publisher.
